# The European carbon cycle response to heat and drought as seen from atmospheric CO_2_ data for 1999–2018

**DOI:** 10.1098/rstb.2019.0506

**Published:** 2020-09-07

**Authors:** C. Rödenbeck, S. Zaehle, R. Keeling, M. Heimann

**Affiliations:** 1 Max Planck Institute for Biogeochemistry, Jena, Germany; 2 Scripps Institution of Oceanography, University of California, San Diego, CA, USA; 3 Institute for Atmospheric and Earth System Research (INAR), Faculty of Science, University of Helsinki, Helsinki, Finland

**Keywords:** drought, net ecosystem exchange, atmospheric inversion, interannual variability

## Abstract

In 2018, central and northern parts of Europe experienced heat and drought conditions over many months from spring to autumn, strongly affecting both natural ecosystems and crops. Besides their impact on nature and society, events like this can be used to study the impact of climate variations on the terrestrial carbon cycle, which is an important determinant of the future climate trajectory. Here, variations in the regional net ecosystem exchange (NEE) of CO_2_ between terrestrial ecosystems and the atmosphere were quantified from measurements of atmospheric CO_2_ mole fractions. Over Europe, several observational records have been maintained since at least 1999, giving us the opportunity to assess the 2018 anomaly in the context of at least two decades of variations, including the strong climate anomaly in 2003. In addition to an atmospheric inversion with temporally explicitly estimated anomalies, we use an inversion based on empirical statistical relations between anomalies in the local NEE and anomalies in local climate conditions. For our analysis period 1999–2018, we find that higher-than-usual NEE in hot and dry summers may tend to arise in Central Europe from enhanced ecosystem respiration due to the elevated temperatures, and in Southern Europe from reduced photosynthesis due to the reduced water availability. Despite concerns in the literature, the level of agreement between regression-based NEE anomalies and temporally explicitly estimated anomalies indicates that the atmospheric CO_2_ measurements from the relatively dense European station network do provide information about the year-to-year variations of Europe’s carbon sources and sinks, at least in summer.

This article is part of the theme issue ‘Impacts of the 2018 severe drought and heatwave in Europe: from site to continental scale’.

## 1. Introduction

The year 2018 saw anomalous heat and drought conditions especially in central and northern parts of Europe over an unprecedented period of time. [1]. Climate anomalies such as these affect the functioning of terrestrial ecosystems, thus causing anomalies in the net ecosystem exchange (NEE) of CO_2_ with the atmosphere through enhancement or suppression of photosynthesis, autotrophic and heterotrophic respiration, biomass burning and mortality [2]. NEE is understood here as the entire CO_2_ exchange between land ecosystems and the atmosphere, including fires. As hot conditions are predicted to become more frequent in the future decades owing to climate change [3], they will likely lead to decadal trends in NEE, which can feed back to the climate trends. A quantitative understanding of the climate effects on NEE is therefore necessary for realistic climate prediction.

Though Europe only accounts for a relatively small part of global NEE, it enjoys one of the densest networks of ecosystem and atmospheric trace gas measurement (including many stations harmonized within the Integrated Carbon Observation System (ICOS)), providing a basis for studying the processes underlying climate–carbon cycle coupling in mid-latitude ecosystems. To this end, climate anomalies like that in 2018 can be employed as ‘natural experiments’. A way to do that is to quantify NEE anomalies and to relate them quantitatively to the underlying climate anomalies. Spatially resolved variations in NEE can be estimated from atmospheric CO_2_ data by inversion of atmospheric transport ([4–8] and many others]). By combining such an atmospheric transport inversion with regression terms expressing interannual NEE anomalies in terms of air temperature anomalies scaled by adjustable NEE-T sensitivity factors, the link between temperature (*T*) and NEE can directly be estimated [9]. Temperature acts as a proxy of climate variations here, representing both direct temperature effects and effects of co-varying climate variables such as water availability and incoming radiation. The regression turned out to be meaningful because the NEE-T sensitivities inferred from this ‘NEE-T inversion’ were found to be consistent with NEE-T regression coefficients calculated from eddy covariance data [9]. Moreover, though all year-to-year NEE variations from the NEE-T inversion originate from temperature variations by construction, they capture a large fraction of the large-scale interannual NEE variations as seen by a ‘standard inversion’ having explicit interannual degrees of freedom, both for tropical and northern extratropical NEE [9].

**Figure 1.**
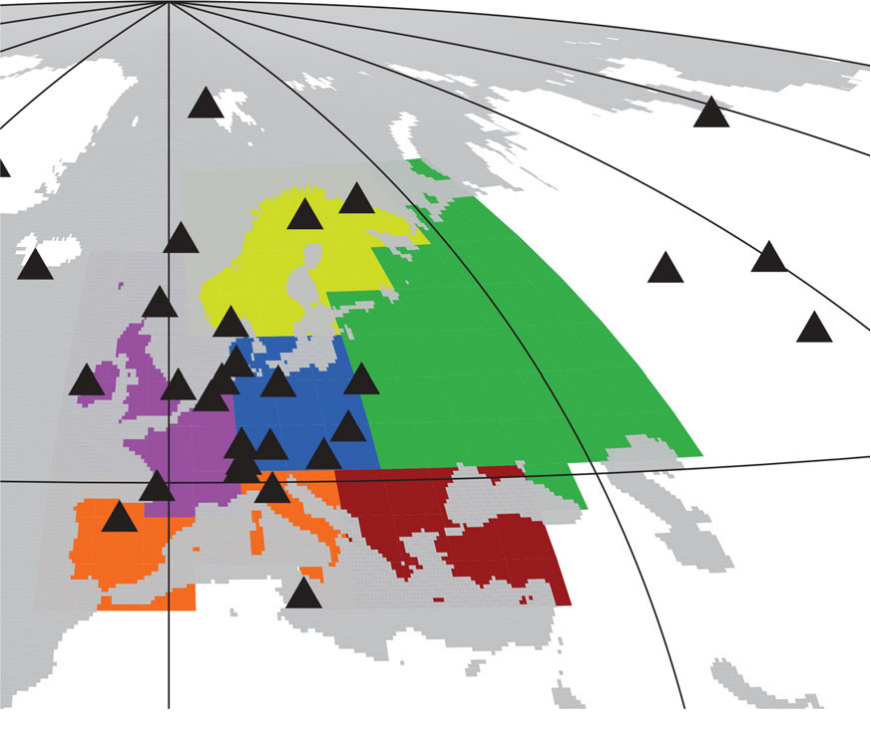
Aggregation regions for time-series plots (colours), and the locations of the atmospheric measurement stations used in some or all inversions (black triangles).

In this contribution to the special issue on the 2018 European heat and drought wave, we compare the NEE response in summer 2018 with summer anomalies during the previous 20 years, in particular, the strong one in 2003 [10]. We use NEE estimates from the Jena CarboScope atmospheric inversion (update of [11]). Though this is a global inversion with a resolution of fluxes and atmospheric transport considerably coarser than in the regional inversions presented by Thompson *et al.* [12], it estimates the NEE history over a longer time frame. The analysis is mostly done for NEE at the spatial scale of European subregions (figure 1) similar to the regions used by the EUROCOM project [13]. Extending the NEE-T inversion [9] outlined above, we introduce an experimental ‘NEE-T-W inversion’ that takes the effect of water availability (*W*) explicitly into account, in addition to temperature. Based on this, we discuss the sensitivity of European summer NEE to heat and drought. We further discuss the limits of inversion-based estimates of year-to-year variations in summer NEE on the spatial scale of the European subregions.

## 2. Methods

### (a) The standard inversion

We estimated spatio-temporal variations of NEE from long-term atmospheric CO_2_ measurements, using a global inversion of atmospheric transport (Jena CarboScope, 8–9], see http://www.BGC-Jena.mpg.de/CarboScope/). We used two of the current CarboScope products (v.4.3) based on differently large sets of atmospheric stations (table 1): the run s99oc_v4.3 uses 50 stations covering all the 1999–2018 analysis period (table 2), thus avoiding spurious year-to-year NEE variations that can arise from starting or ending observational records; the run s10oc_v4.3 uses a larger set of 70 stations, including several additional European ones for better resolving detailed signals, however only covering 2010–2018.

**Table 1.** Inversion runs used in this study.

kind of inversion	specific feature (if any)	no. atm. stations (globally) (in/around Europe)		period of validity	CarboScope run ID
*base cases of this study*					
standard		50	9	1999–2018	s99oc_v4.3
standard		70	22	2010–2018	s10oc_v4.3
NEE-T		95	28	1957–2018	sEXT10ocNEET_v4.3
NEE-T-W		95	28	1957–2018	sEXT10ocNEETW_v4.3
*test cases around base standard inversion s99oc_v4.3*					
standard	half *a priori* uncertainty	50	9	1999–2018	s99oc_tight_v4.3
standard	double *a priori* uncertainty	50	9	1999–2018	s99oc_loose_v4.3
standard	shorter spatial correlations	50	9	1999–2018	s99oc_short_v4.3
standard	shorter temporal correlations	50	9	1999–2018	s99oc_fast_v4.3
*test cases around base NEE-T-W inversion sEXT10ocNEETW_v4.3*					
NEE-T-W		50	9	1957–2018	s99ocNEETW_v4.3
NEE-T-W		70	22	1957–2018	s10ocNEETW_v4.3
NEE-T-W	half *a priori* uncertainty	95	28	1957–2018	sEXT10ocNEETW_tight_v4.3
NEE-T-W	double *a priori* uncertainty	95	28	1957–2018	sEXT10ocNEETW_loose_v4.3
NEE-T-W	longer spatial correlations^a^	95	28	1957–2018	sEXT10ocNEETW_long_v4.3
NEE-T-W	additional explanatory	95	28	1957–2018	sEXT10ocNEETWTTWW_v4.3
	variables^b^: Δ*T*^2^, SPEI06^2^				
NEE-T-W	additional explanatory	95	28	1957–2018	sEXT10ocNEETWTTWWTW_v4.3
	variables^b^: Δ*T*^2^, SPEI06^2^,				
	Δ*T* · SPEI06				

^a^Longer spatial correlations in the regression terms.

^b^In addition to the explanatory variables Δ*T* (temperature anomaly) and SPEI06 present in all NEE-T-W inversions. The regression terms of all explanatory variables are normalized identically (see §2c)

**Table 2.** Atmospheric measurement stations in Europe and surrounding oceans (the inversions use further stations around the world).

station code	institution^a^	record type^b^	available regular data period^c^	used for:		
				s99	s10	sEXT10
CMN	CNR-ISAC	n	1979.4–present	yes	yes	yes
IZO	AEMET	h	1984.5–present	yes	yes	yes
SSL	UBA	n	1988.0–present	yes	yes	yes
MHD	NOAA	f	1991.5–present	yes	yes	yes
SIS	CSIRO, BGC	f	1992.9–present	yes	yes	yes
HUN	NOAA	f	1993.2–present	yes		
HUN115	HMS^d^	d	1994.8–present		yes	yes
ZEP	NOAA	f	1994.2–present	yes	yes	yes
AZR	NOAA	f	1995.0–present	yes	yes	yes
WIS	NOAA	f	1996.0–present	yes	yes	yes
KAS	AGH^d^	n	1996.6–present		yes	yes
PAL	FMI, NOAA	d, f	1998.5–present	yes	yes	yes
CBW207	TNO^d^	d	2000.3–present		yes	yes
SUM	NOAA	f	2003.5–present		yes	yes
JFJ	EMPA, BGC	n, f	2005.0–present		yes	yes
BIK300	BGC	d, f	2005.9–present		yes	yes
HPB	NOAA	f	2006.3–present		yes	yes
LUT	CIO-RUG^d^	d	2006.4–present		yes	yes
LMP	NOAA	f	2006.8–present		yes	yes
WAO	UEA^d^	d	2007.9–present		yes	yes
PRS	RSE^d^	n	2008.0–present		yes	yes
BIR	NILU^d^	d	2009.7–present		yes	yes
BIS	LSCE^d^	d	2009.8–present		yes	yes
CIB	NOAA	f	2009.4–present			yes
STM	NOAA	f	1981.3–2009.9			yes
WES	UBA	d	with longer gaps			yes
ICE	NOAA	f	with longer gaps			yes
NGL	UBA	d	1994.0–2013.9			yes
TER	MGO	f	1999.1–2017.8			yes

^a^AEMET, Izaña Atmospheric Research Center, Meteorological State Agency of Spain [14]; AGH, University of Science and Technology, Poland; BGC, Max Planck Institute for Biogeochemistry, Germany [15]; CIO-RUG, Centre for Isotope Research, Rijksuniversiteit Groningen, The Netherlands; CNR-ISAC, Italian Air Force Meteorological Service, Institute of Atmospheric Sciences and Climate [16]; CSIRO, Commonwealth Scientific and Industrial Research Organisation, Australia [17]; EMPA, Swiss Federal Laboratories for Materials Science and Technology; FMI, Finnish Meteorological Institute [18]; HMS, Hungarian Meteorological Service [19]; LSCE, Laboratoire des Sciences du Climat et de l’Environnement, France [20]; MGO, Voeikov Main Geophysical Observatory, Russian Federation (http://voeikovmgo.ru/index.php?lang=en); NILU, Norwegian Institute for Air Research; NOAA, National Oceanic and Atmospheric Administration/Earth System Research Laboratory, USA [21]; RSE, Ricerca sul Sistema Energetico, Italy; TNO, Netherlands Organisation for Applied Scientific Research; UBA, Umweltbundesamt, Germany [22]; UEA, University of East Anglia, UK.

^b^d, *in situ*, day-time selected; f, flask; h, *in situ*, all hours; n, *in situ*, night-time selected.

^c^The number following the dot in the dates (e.g. in 1979.4) gives the decimal fraction of the year.

^d^Data taken from the compilation prepared by the Drought 2018 Team [23].

All inversions used in this paper only optimize land CO_2_ fluxes, while fossil fuel emissions and ocean CO_2_ fluxes are prescribed [9]. Atmospheric transport is simulated by the TM3 model [24] on a spatial resolution of 5° longitude and *ca* 4° latitude, driven by NCEP reanalysis meteorological fields [25]. The flux fields and all sets of degrees of freedom are numerically resolved on the same spatial resolution.

### (b) The NEE-T inversion

While the ‘standard inversion’ of §2a directly estimates the interannual variations of NEE from the atmospheric CO_2_ signals, the NEE-T inversion instead effectively performs a linear regression of interannual NEE anomalies against interannual anomalies of air temperature (see [9] for details). This is done by using spatially and seasonally explicit regression coefficients as adjustable degrees of freedom. These coefficients (***γ***_NEE-T_) are identical in each year (during the ‘period of validity’, here 1957–2018), but are allowed to vary smoothly both seasonally (with a correlation length of about three weeks) and spatially (with correlation lengths of about 1600 km in the longitude direction and 800 km in the latitude direction). The ***γ***_NEE-T_ degrees of freedom have prior values of zero, and *a priori* uncertainties scaled such that the *a priori* uncertainty of the global July integral of the regression term (averaged over all Julys of the ‘period of validity’) is identical to the corresponding uncertainty of the explicit interannual term of the standard inversion (July is an arbitrary choice, in line with the normalization with respect to the flux at the middle of the final year used in CarboScope so far).

Formally, the estimated ***γ***_NEE-T_ represent the local and season-specific sensitivities of NEE to interannual variations in temperature, but include the sensitivities to other climate variables covarying with temperature. The NEE-T inversion is considerably more strongly regularized than the standard inversion, because the regression term involving only 13 temporal degrees of freedom (Fourier modes) per land pixel replaces the explicit interannual term of the standard inversion having 1320 temporal degrees of freedom (Fourier modes) per land pixel.

The NEE-T inversion is constrained by an ‘extended’ set of stations (table 2), including ones not covering all the analysis period. This is possible in the NEE-T inversion since the regression uses the same degrees of freedom repeatedly each year and thus is not very sensitive to changes in the station network (see [9, §2.2]). Using an extended station set potentially provides higher spatial resolution than the standard inversion. The specific NEE-T inversion run presented here uses the set ‘sEXT10’ of 95 stations, including all of the set ‘s10’ used in one of the standard inversions (table 2).

While the updated NEE-T inversion (the current CarboScope product sEXTocNEET_v4.3) includes a relaxation term not yet present in Rödenbeck *et al.* [9], we again dropped this relaxation term in the NEE-T inversion runs used in this paper, because it would have complicated the analysis of the amplitudes of the two regression terms in the NEE-T-W inversion described below, while its influence on the European NEE variations is small anyway.

### (c) The NEE-T-W inversion

In order to address the respective roles of heat and drought, this study introduces an experimental NEE-T-W inversion as a multivariate extension of the NEE-T inversion. In addition to the regression term against temperature, the NEE-T-W inversion also contains a regression term against water availability, here represented by the six-monthly accumulated Standardized Precipitation Evapotranspiration Index (SPEI06, update of [26], accessed from spei.csic.es/map/maps.html on 4 October 2019). SPEI06 represents the climatic water balance (precipitation minus evapotranspiration) accumulated over the past six months. In the version used here, evapotranspiration has been calculated by the Thornthwaite equation [27]. The SPEI06 time series has been standardized assuming a log-logistic probability density function.

The water availability term has the same structure as the temperature term, involving independent sensitivity coefficients (***γ***_NEE-W_) with an *a priori* correlation structure identical to that of ***γ***_NEE-T_. Owing to the uncertainty scaling described in §2b, temperature and water availability have the same weight in the NEE-T-W inversion, at least at global scale in summer.

## 3. Results

### (a) Which years saw anomalous summer NEE in Europe during 1999–2018?

**Figure 2.**
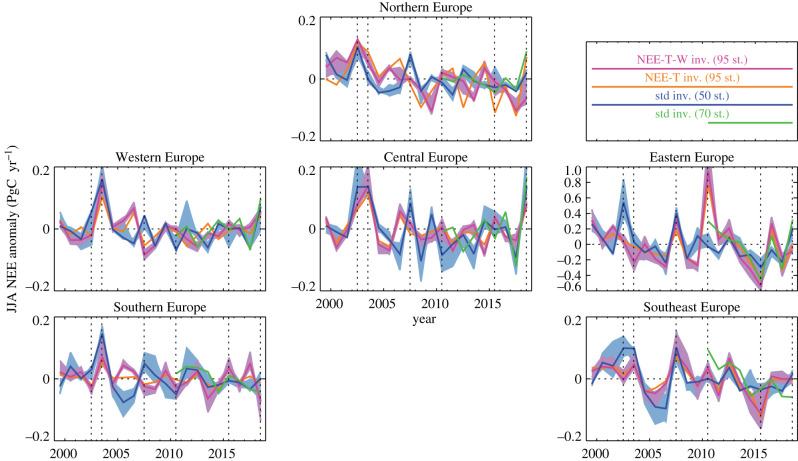
Time series of the estimated summer (June–July–August, JJA) CO_2_ flux anomalies in Europe divided into the six geographical regions depicted in figure 1. The shaded areas of corresponding colour around the NEE-T-W inversion (magenta) and the longer standard inversion (blue) comprise the results of respective sets of sensitivity cases (table 1). Vertical lines mark the summers of 2002, 2003, 2007, 2010, 2015 and 2018 considered in figure 3. Note the wider vertical range in the Eastern Europe panel, needed owing to the larger area of this region. inv., inversion; st., stations; std, standard.

In Western Europe, the summer of 2018 is estimated to have seen the second largest positive NEE anomaly after the summer of 2003 (figure 2), both by the standard inversion with explicit intrannual degrees of freedom (blue) and the NEE-T-W inversion relating NEE anomalies to climate anomalies (magenta). In contrast to the 2018 anomaly, the 2003 anomaly also occurs in Southern Europe.

In Central Europe, the standard inversion sees the 2018 anomaly about as large or even larger than that in 2003, while another anomaly of similar size is detected for 2002, as well as further positive and negative excursions of smaller size. The NEE-T-W inversion sees the summers of 2003 and 2018 as similarly anomalous to the standard inversion, but disagrees on all the smaller excursions.

In Northern Europe, a similar level of agreement between the standard and NEE-T-W inversions is found, though the largest summer anomaly is detected by all inversions in 2002. For 2018, only the standard inversion with the larger station set (green) and the NEE-T inversion see an appreciable anomaly here.

Going on to Eastern Europe, the rank of 2018 further declines. The largest anomaly here is estimated by the NEE-T-W inversion for the ‘fire summer’ of 2010. This anomaly is not seen in the standard inversion s99oc_v4.3 (with longer period but fewer stations, blue), but the additional stations used by s10oc_v4.3 (green)—in particular, Eastern European stations such as BIK and KAS (table 2)—reveal an anomaly in the summer of 2010 even without the help of the climate data. Conversely, the anomaly in 2002 extends into Eastern Europe in the standard inversion, while the NEE-T-W inversion does not put any anomaly in that summer. Qualitative agreement between the two types of inversion is seen for 2007 and 2015–2018, where the quantitative agreement again substantially improves from using more stations (green versus blue). Note that the larger flux amplitude in Eastern Europe is only due to its larger area, while the per-area fluxes of all the regions are quite comparable.

### (b) How were the strongest summer NEE anomalies distributed spatially?

**Figure 3.**
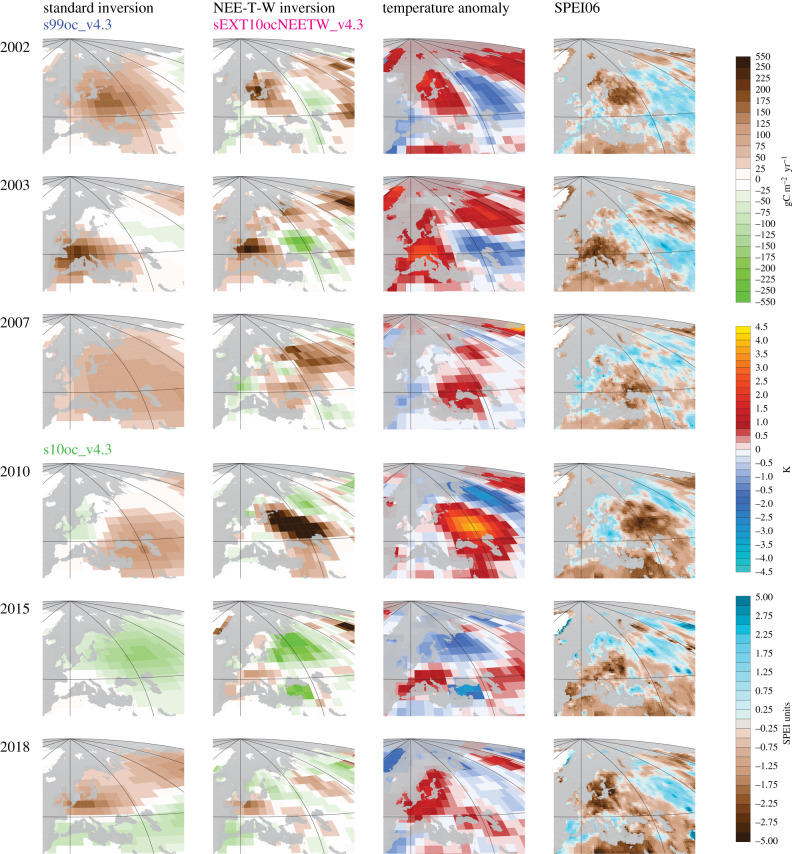
NEE anomalies estimated for the summers of 2002 (top), 2003, 2007, 2010, 2015 and 2018 (bottom) by the standard inversion (left) and the NEE-T-W inversion (sEXT10ocNEETW_v4.3, 2nd from left). For standard inversion, we used s99oc_v4.3 in the years before 2010, and s10oc_v4.3 from 2010 onwards. The maps show the deviations of summer (June–July–August, JJA) NEE from their 1999–2018 mean over Europe and surroundings in ${\text{gC m}}^{-2}\;{\text{yr}}^{-1}$ (note the unequal spacing of the colour bar to accommodate very high and low values). For comparison, summer (JJA) anomalies of temperature (2nd from right) and the August value of the six-monthly Standardized Precipitation Evapotranspiration Index (SPEI06 [25], right) are given.

The time-series view is corroborated by the spatial patterns of the most pronounced summer NEE anomalies (figure 3). For example, standard and NEE-T-W inversions (left two columns) agree quite well on where the respective centres of the 2003 and 2018 anomalies are located, with the centre of the 2018 flux anomaly (bottom row) more to the northeast of that of the 2003 flux anomaly (2nd row of maps from top). In 2015, both inversions estimate anomalously high uptake in northern Russia (figure 3); west of that, both inversions only estimate smaller anomalies (almost no anomaly in the standard inversion and a slight release anomaly in the NEE-T-W inversion). For 2010, both inversions agree on a mainly neutral flux in Western and Central Europe (figure 3). The NEE-T-W inversion predicts a strong dipole of outgassing in Russia further south and uptake further north, which is again averaged into a more widespread but weaker outgassing by the standard inversion.

By construction, the NEE-T-W inversion gives NEE anomaly patterns closely related to the temperature anomaly or SPEI06 patterns (right columns). In general, these NEE anomaly patterns are more structured than those of the standard inversion, especially in the eastern part of Europe were atmospheric CO_2_ data coverage is lower (figure 1).

### (c) How are the interannual summer NEE anomalies related to climate anomalies?

**Figure 4.**
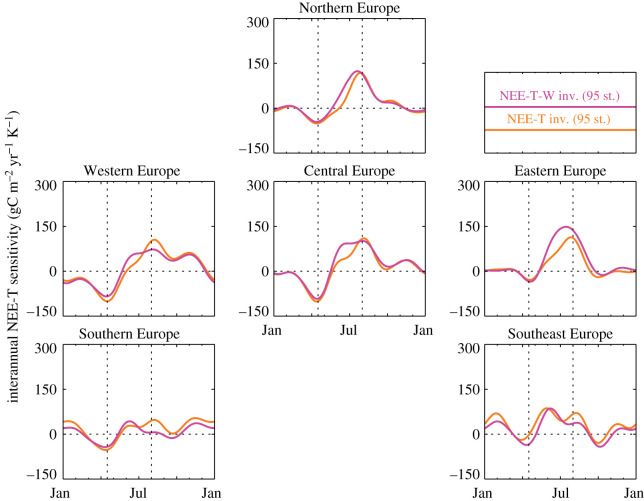
Estimated sensitivity ***γ***_NEE-T_ of NEE to interannual temperature variations, averaged over the regions of figure 1, plotted against the climatological month. Note that it is a univariate sensitivity (i.e. it includes effects of other climate variables covarying with temperature) in the NEE-T inversion but a multivariate one in the NEE-T-W inversion. inv., inversion; st., stations.

**Figure 5.**
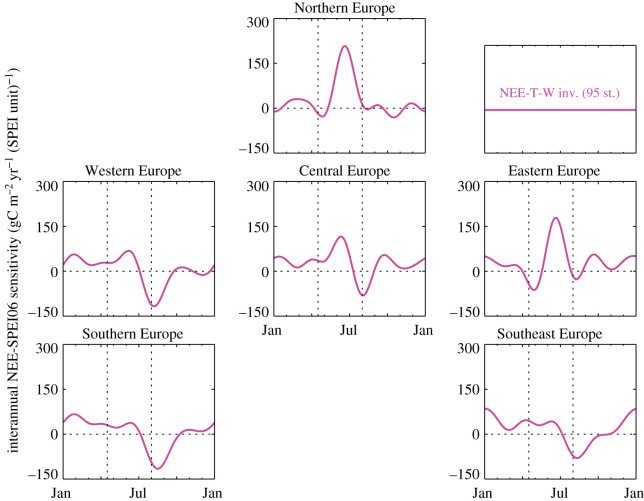
Estimated sensitivity $\gamma_{\mathrm{NEE}\text{-}W}$ of NEE to interannual SPEI06 variations, averaged over the regions of figure 1 plotted against the climatological month. inv., inversion; st., stations.

Figure 4 gives the estimated sensitivity ***γ***_NEE-T_ of NEE to interannual temperature variations. Reflecting that ***γ***_NEE-T_ can vary spatially and with season, we plotted its averages over the European subregions as time series over a climatological year. Importantly, in the univariate NEE-T inversion (orange), ***γ***_NEE-T_ represents an ‘interannual climate sensitivity’ involving both direct temperature effects on NEE and effects from covarying climate variables, while in the multivariate NEE-T-W inversion (magenta) part of the covarying effects are moved to the SPEI06-dependent term. Nevertheless, ***γ***_NEE-T_ is similar between the NEE-T and NEE-T-W inversions, with the general pattern of negative sensitivity in spring and positive sensitivity in summer (as in [9]). In Southern and Southeast Europe, the ‘multivariate’ ***γ***_NEE-T_ is slightly lower than the ‘univariate’ one in late summer, while in all the rest of Europe, the multivariate ***γ***_NEE-T_ is slightly higher in early summer. Corresponding to this tendency, we find a compensating positive sensitivity ***γ***_NEE-W_ against interannual variations of SPEI06 (which is roughly anti-correlated with temperature) in the more northern parts of Europe in early summer, and negative ***γ***_NEE-W_ in the more southern parts in late summer (figure 5).

**Figure 6.**
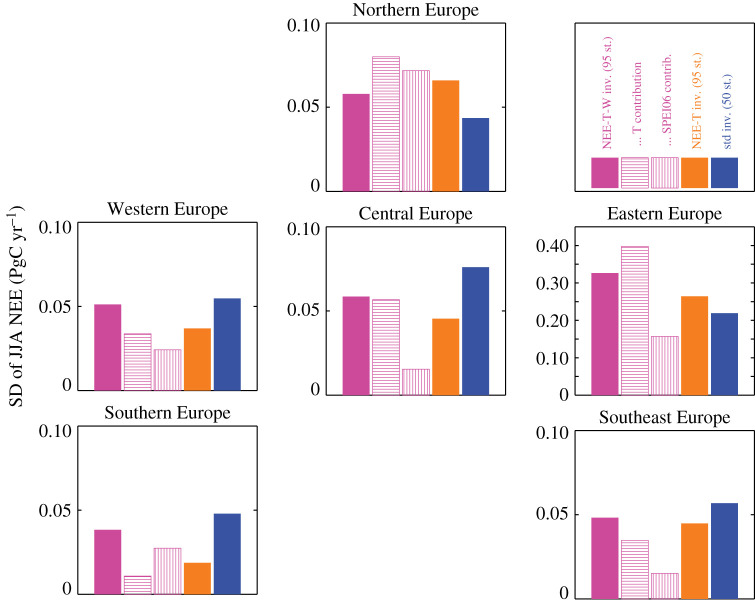
Amplitudes (expressed as temporal standard deviations 1999–2018) of estimated interannual variability in summer NEE. Solid bars give the amplitudes of the total variability in summer NEE from the three inversions covering the analysis period completely. Hatched bars give amplitudes of the components related to temperature and SPEI06 variations in the NEE-T-W inversion (magenta); note that these do not necessarily add up to the total amplitude owing to mutual (anti-) correlations. inv., inversion; st., stations; std, standard.

The respective magnitudes of the corresponding temperature-related or SPEI06-related NEE variations are shown as hatched bars in figure 6. The estimated relative amplitudes of summer NEE variations are distinctly different between the European subregions: while in Western Europe the temperature-related contribution (horizontally hatched) and the SPEI06-related contribution (vertically hatched) both have about half the amplitude of the total summer NEE variations (solid bar), Central Europe is estimated to be clearly dominated by temperature, and Southern Europe dominated by SPEI06. In Northern Europe, both contributions have larger amplitudes than the total variations, which is possible because they are strongly anti-correlated and thus partially compensating each other. The anti-correlations indicate that the two contributions are not independently constrained in Northern Europe, which likely also applies to a lesser degree to the other regions.

### (d) How much signal is shared between climate-dependent and temporally explicit NEE estimates?

**Figure 7.**
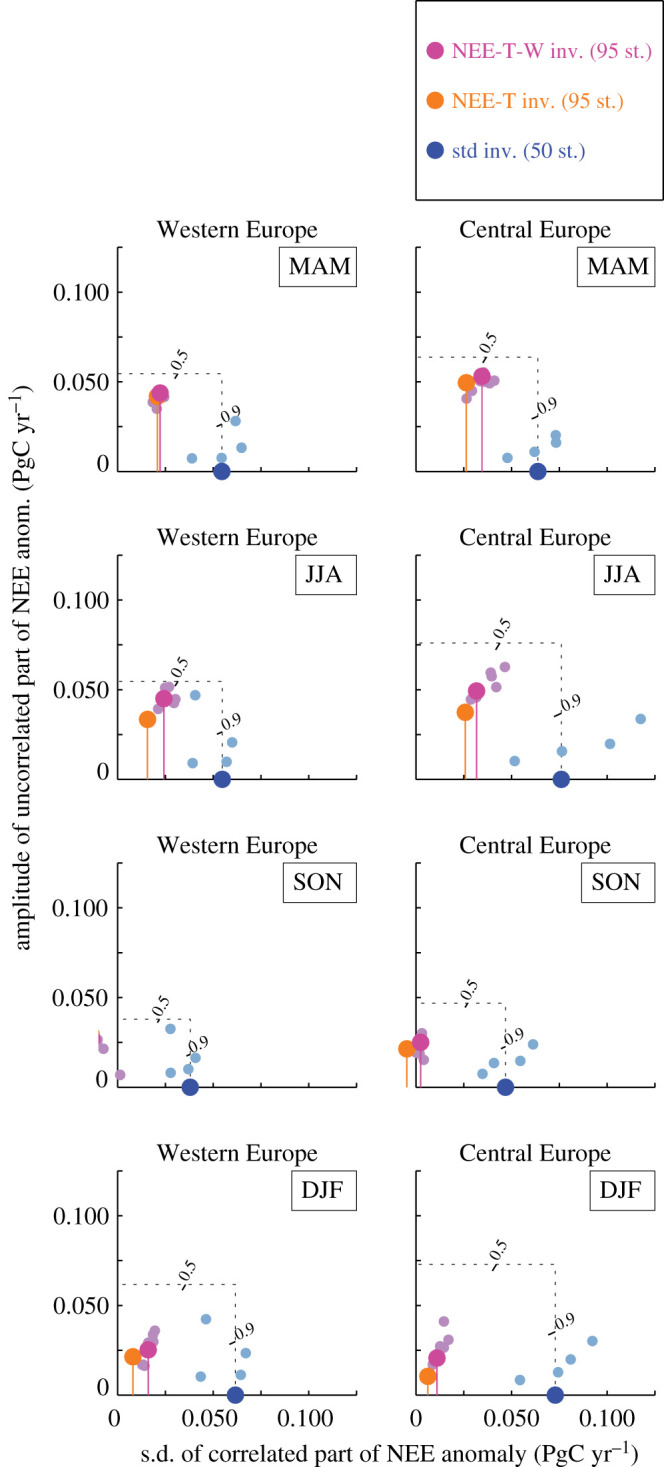
Agreement of the NEE-T inversion (orange) and the NEE-T-W inversion (magenta) with the standard inversion (blue) for year-to-year variations of NEE in spring (March–April–May, top), summer (June–July–August), autumn (September–October–November) and winter (December–January–February, bottom). The agreement is quantified in terms of Taylor plots [27]: The horizontal position of a dot gives the amplitude (temporal standard deviation) of the time-series portion correlated with the standard inversion, while the vertical position (length of the stem) gives the amplitude of the uncorrelated portion. Note that the standard inversion (blue) is *not* meant to represent the ‘truth’ here (even though it reflects the measured atmospheric signals most directly), i.e. the Taylor plots in this figure cannot be read as a measure of performance for the NEE-T or NEE-T-W inversions, because the inversions are all expected to carry specific errors (see discussion in the text). The smaller pale dots correspond to the test cases (as the shaded areas in figure 2). inv., inversion; st., stations; std, standard.

Figure 7 quantifies how the NEE-T and NEE-T-W inversions agree with the standard inversion (using the longer standard inversion s99oc_v4.3 covering all the analysis period), for the Western and Central Europe regions where the highest density of measurement stations is found. In the Taylor diagrams [28] shown, the standard inversion formally takes the role of the reference, even though it cannot necessarily be considered more authoritative (see §4b).

In summer (June–July–August, JJA, 2nd row of panels from top), the NEE-T inversion (orange) contains the variations seen by the standard inversion with about a third of their amplitude (horizontal position with respect to the blue dot), but also variability uncorrelated to the standard inversion with an amplitude of about half that of the standard inversion result (vertical position). This results in a correlation coefficient (represented in the Taylor diagrams in the angle coordinate) of slightly more than 0.5 in both regions. Compared with the NEE-T inversion, the NEE-T-W inversion (magenta) contains a larger share of the standard inversion result (about half). Since it also contains larger uncorrelated signals, however, its correlation coefficient is similar.

In spring (March–April–May, MAM, top row), the situation is quite similar to that in summer. In winter (December–January–February, DJF, bottom row), the fractions of the correlated and uncorrelated variations are both lower than in summer. The NEE-T-W inversion again shares a larger fraction of the variations that the standard inversion sees. In autumn (September–October–November, SON, 2nd row from bottom), NEE-T and NEE-T-W inversions are uncorrelated or even slightly anti-correlated to the standard inversion, i.e. they do not share any signal. However, autumn also has the smallest amplitude of variations among all seasons anyway.

## 4. Discussion

### (a) Which processes can explain the *γ*_NEE-T_ and *γ*_NEE-W_ found?

In spring, the NEE-T inversion estimated the sensitivity ***γ***_NEE-T_ of NEE to interannual variations in temperature (*T*) to be negative throughout Europe (figure 4, orange) and also throughout all the northern extratropics (not shown). Considering that in the univariate NEE-T analysis, *T* also acts as proxy for variations in any covarying climate variable, Rödenbeck *et al.* [9] gave a possible underlying causation that we write here as

4.1 $$\begin{array}{ccccc} \underline{⊕\;T} & \Rightarrow & \\ ⇕ & & ⊕\;\text{GPP} & \Rightarrow & \underline{⊖\;\text{NEE}},\\ ⊕\;\text{radiation} & \Rightarrow & \end{array}$$

where ⊕ means ‘higher than usual’, ⊖ means ‘lower than usual’, ⇒ means ‘causes’, and ⇔ means ‘tends to coincide with’. For better orientation, the underbars highlight the two quantities whose correlation is being considered. NEE follows the usual sign convention, where lower values mean less CO_2_ release or more CO_2_ uptake. In summer (when—in contrast to spring—photosynthesis is not *T*-limited any more but water-limited), ***γ***_NEE-T_ has been found to be positive, which can arise from

4.2 $$\underline{⊕\;T}\Rightarrow ⊕\;\text{respiration}\Rightarrow \underline{⊕\;\text{NEE}},$$

but also from

4.3 $$\underline{⊕\;T}\Leftrightarrow ⊖\;\text{water availability}\Rightarrow ⊖\;\text{GPP}\Rightarrow \underline{⊕\;\text{NEE}}.$$

In the multivariate NEE-T-W inversion, the dependence of NEE on water availability is—at least formally—handled explicitly through the regression against SPEI06. For the sensitivity $\gamma_{\mathrm{NEE}\text{-}W}$ of NEE against interannual variations in SPEI06, we found negative values in mid-latitudes in late summer (figure 5, lower panels). Such negative sensitivities can arise from

4.4 $$\underline{⊕\;\text{SPEI}}\Rightarrow ⊕\;\text{GPP}\Rightarrow \underline{⊖\;\text{NEE}}.$$

Indeed, this may replace the indirect causation of equation (4.3). To the extent that the NEE-T-W inversion realistically separates the respective influences of temperature and water availability (see discussion in §4b below), the large relative amplitude of the water-related component in Southern Europe (figure 6) may indicate a dominance of drought-inhibited photosynthesis according to equation (4.4), while the large relative amplitude of the temperature-related component in Central Europe (figure 6) tends to favour heat-stimulated ecosystem respiration according to equation (4.2).

Note that a positive ***γ***_NEE-T_ would also arise via changes in vapour pressure deficit (VPD),

4.5 $$\underline{⊕\;T}\Rightarrow ⊕\;\text{VPD}\Rightarrow ⊖\;\text{GPP}\Rightarrow \underline{⊕\;\text{NEE}},$$

which would represent a third possible pathway besides equation (4.2) or equation (4.4). However, the dependence of VPD on temperature only dominates the short-term responses, while the regression done here is only sensitive to relationships on about monthly and longer-term time scales. We therefore assume that equation (4.5) is not a dominant pathway.

We further note that ecosystem respiration has been found to correlate (positively) with GPP [29]. This has the potential to partially cancel the effect of GPP variations on NEE variations. In cases nevertheless pointing to equation (4.4) as the dominant process (Southern Europe), the possibility of a partially cancelled GPP influence would strengthen this process attribution. However, in cases where equation (4.4) does not seem to play a dominant role (Central Europe), we cannot exclude that important GPP variability does exist but gets reduced owing to the opposing ecosystem respiration variability.

In the high latitudes, we found positive $\gamma_{\mathrm{NEE}\text{-}W}$ values in early summer (figure 5, upper panels). Even though high-latitude GPP is not water-limited, a positive $\gamma_{\mathrm{NEE}\text{-}W}$ can arise indirectly from radiation limitation according to

4.6 $$\begin{array}{ccccc} ⊕\;\text{cloud-cover} & \Rightarrow & ⊕\;\text{precipitation} & \Rightarrow & \underline{⊕\;\text{SPEI}}\\ ⇓ & \\ ⊖\;\text{radiation} & \Rightarrow & ⊖\;\text{GPP} & \Rightarrow & \underline{⊕\;\text{NEE}}. \end{array}$$

As the NEE-T-W inversion was not able to separate temperature and water-related effects in Northern Europe (§3c), we cannot make any statements on the relative weights of the alternative causations equations (4.2) or (4.6).

The response of NEE to climatic variations and anomalies is very likely also related to other causations. Given their limitations discussed below, the results of the NEE-T-W inversion certainly cannot uniquely identify the roles of all such contributions, but do indicate that the ones highlighted here may be among the dominating ones.

### (b) How trustworthy are the estimated summer NEE anomalies within Europe?

On the spatial scale of European subregions, the standard inversion (directly reflecting the signals in atmospheric CO_2_ data, blue) and the NEE-T or NEE-T-W inversions (deriving all NEE variations from temperature or SPEI06 variations, magenta) share many of the larger anomalies in summer (§3, figure 2), although the linear correlation is only moderate (figure 7). Though this level of agreement is weaker than that found for annual NEE in the large-scale $90^{\circ }\text{N}-25^{\circ }\text{N}$ and $25^{\circ }\text{N}-90^{\circ }\text{S}$ latitudinal bands ([9], NEE-T inversion only), it is notable that some agreement is still found also for the small spatial scale of European subregions. It suggests that the sustained atmospheric CO_2_ data records from a relatively dense network of stations indeed contain information about year-to-year variations of summer NEE in Europe. This conclusion is based on the facts that

(i) the NEE-T and NEE-T-W inversions have much fewer degrees of freedom than the standard inversion and can only produce NEE variations already contained in the temperature or SPEI06 fields, while
(ii) the similarities of the NEE-T and NEE-T-W inversions to the standard inversion have been achieved by sensitivities ***γ***_NEE-T_ and $\gamma_{\mathrm{NEE}\text{-}W}$ that are meaningful themselves, in the sense that
  • they are compatible (within sizeable uncertainties) with regressions of the fully independent eddy covariance data (to essentially the same degree as shown in [9]), and
  • they show seasonal and large-scale spatial patterns accessible to interpretation in terms of ecological processes (§4a).

The conclusion that the atmospheric CO_2_ data do contain interannual NEE signals is further underlined by the finding that using more stations in the standard inversion helps to increase its agreement with the NEE-T and NEE-T-W inversions (seen especially in Eastern Europe, §3a). Further support comes from the fact that the NEE estimates from the standard inversion are, when averaged over the regional scale, already correlated with the (independent) temperature anomalies (though of course less strongly than those of the NEE-T inversion, not shown).

Analysing a regional inversion over Europe up to 57.5°N and 22.5°E (approximately Western, Southern and Central Europe of figure 1) and the period 2002–2007, Broquet *et al.* [30, p. 9053] warned that year-to-year variations in monthly NEE (i.e. NEE of a given month each year) may be too small to ‘be analysed safely’, because they found their temporal standard deviation (signal) to be smaller than their *a posteriori* uncertainties (noise), especially for the winter months. In some summer months, however, they also found signal-to-noise ratios slightly above unity. The comparison of standard and NEE-T-W inversions discussed above adds a new piece of evidence suggesting that the situation is somewhat more optimistic than concluded by Broquet *et al.* [30], maybe also owing to our longer analysis period. Consistent with the seasonality of signal-to-noise ratios found by Broquet *et al.* [30], we also find the best agreement between standard and NEE-T-W inversions in summer, and less good in autumn and winter (figure 7).

Nevertheless, we expect the flux and sensitivity estimates to carry substantial errors, though these are difficult to quantify. Part of the errors is indicated by the range of results from test runs where *a priori* correlation lengths, *a priori* uncertainty ranges, or the set of explanatory variables have been changed within the limits deemed reasonable (shadings around the standard and NEE-T-W inversions in figure 2). In general, the range across these test results (noise) is smaller than the year-to-year variations (signal). As the set of test runs includes regressions also using quadratic explanatory variables, our results do not seem to be limited by nonlinearities in the NEE responses. Note that the location of NEE anomalies estimated by the standard inversion may be sensitive to the chosen correlation lengths, shifting features between the European subregions of figure 1. Owing to this, NEE summed over several subregions (e.g. the sum of Western and Central Europe, not shown) can be much better constrained than for the regions individually.

Is the standard inversion or the NEE-T-W inversion more realistic? Concievably, both types of inversion carry errors that are partially complementary: the standard inversion misses parts of the variability not seen by any station (as illustrated by the differences arising when more stations are added), while the NEE-T and NEE-T-W inversions miss any NEE variability not correlated to momentary *T* or SPEI06 variations (or, worse, may alias it into wrong locations). Moreover, interannual transport model errors affect the standard inversion directly, but the NEE-T and NEE-T-W inversions only if correlated to *T* or SPEI06 variations. In any case, however, the existing similarities between the standard and NEE-T-W inversions (figures 2 and 7) discussed above suggest that these errors are smaller than the actual NEE variability. As also the standard inversion results are correlated to temperature on the regional scale, we may assume that the more pronounced spatial structure induced by temperature and SPEI in the NEE-T-W inversion results is more realistic than the more spread-out patterns in the standard inversion. The similarities between the two types of inversion further allow the conclusion that a sizeable part of year-to-year variations in summer NEE can be described as a response to momentary climate conditions.

Can the analysis of the model-data residuals provide further insight into whether the standard inversion or the NEE-T-W inversion is more realistic? All inversions used here fit the individual atmospheric CO_2_ data closely. However, only a small fraction of the atmospheric signal even at the European stations is actually related to the interannual variations of European NEE, while most of the atmospheric signal is related to the hemispheric flux seasonality, to the El Niño-dominated global interannual flux variations, and to atmospheric transport variability. When we try to extract the Europe-related signals (by subtracting a global background and interannually filtering the measured and modelled atmospheric CO_2_ mole fraction time series), the standard inversion achieves a somewhat better fit to the atmospheric CO_2_ data than the NEE-T or NEE-T-W inversions (not shown), which however is expected because the standard inversion has many more adjustable degrees of freedom available: lacking a calibration, it is difficult to draw any more quantitative conclusions from the goodness of fit. We further need to acknowledge that any ad hoc extraction of Europe-related signals without explicit consideration of transport is inappropriate because the flux-related signals are not to be expected at individual locations directly but rather in station-to-station differences, which however are related to the fluxes in highly non-trivial ways owing to the substantial temporal variation in transport pathways and to the atmospheric mixing. Therefore, while the analysis of model-data residuals can be useful in many other applications of inverse methods, it does not help for atmospheric inversions, unfortunately: the analysis of residuals in mole fraction space does not provide any information beyond our comparison of the standard and NEE-T-W inversions in terms of their flux estimates, as discussed above. For the same reason, our flux comparison of the standard inversion using 50 stations with that using 70 stations is more informative than a mole fraction comparison with the additional stations used as independent data would be.

In almost all the anomalous years within the last 20 years, anomalies in *T* and SPEI06 are almost always co-located (figure 3, right columns). This raises the question of how the NEE-T-W inversion would actually be able to statistically disentangle the relative roles of heat and drought as causes of the NEE anomalies. However, the six-monthly accumulated SPEI06 changes more gradually than the monthly temperature (not shown); this leads to a partial de-correlation of the two explanatory variables in time and may allow the inversion to differentiate between them. Though such a difference in time scale is plausible also for the respective effects of heat and drought on ecosystems, it remains open as to how appropriate the time evolution of the explanatory variables in the linear NEE-T-W inversion actually is. An inappropriate time evolution has the potential to affect the magnitude and seasonal variation of the sensitivities. While the estimated seasonal patterns of the SPEI06 sensitivity $\gamma_{\mathrm{NEE}\text{-}W}$ (including their differences between high latitudes and mid-latitudes) are ecologically plausible (§4a), an independent verification like that of the temperature sensitivities ***γ***_NEE-T_ based on eddy covariance data [9] is more difficult because SPEI06 values are not available in the FLUXNET2015 dataset.

By construction, the results of the NEE-T-W inversion decidedly depend on the dataset used as explanatory variable in the water availability term. The six-monthly Standardized Precipitation Evapotranspiration Index (SPEI06, §2c) was chosen here because (1) it is based on observations, (2) it was found to match soil-moisture anomalies from satellite-based data well [31] and (3) it is available until the end of our analysis period in 2018. Note that this SPEI dataset uses the Thornthwaite estimation of potential evapotranspiration [27], considered less reliable than the FAO-56 Penman–Monteith estimation [32] used in the alternative ‘SPEIbase’ dataset; however, the latter is currently only available until 2015. There would have been further potential alternative choices, including soil moisture from re-analysis fields or satellite-based soil moisture. A systematic comparison of NEE-T-W inversions using such alternatives is a necessary next step.

### (c) How anomalous was NEE in 2018, compared with previous anomalous years?

The summers of 2003 and 2018 show the largest NEE anomalies within our 20-year analysis period in Western and Central Europe (figure 2). Summed over these two regions, the 2018 summer NEE anomaly is estimated by our ensemble of inversions (all of table 1 except s10oc_v4.3) to be between 42 and 75% of that in 2003. That is, the more intense heat and drought conditions during summer in 2003 seem to outweigh possible legacy effects from the already dry spring on the summer NEE anomaly in 2018.

While the NEE-T-W inversion agrees relatively closely to the standard inversion for the summer 2003, it remains lower than the standard inversion for the summer 2018 (figure 2). This may indicate that the ecosystem response to the anomalous climate was actually stronger in 2018 than in 2003, while the NEE-T-W inversion assumes the same sensitivity each year. Indeed, Buras *et al.* [1] reported significantly larger responses of satellite-based vegetation indices to the climatic water balance in 2018 compared with 2003. In addition to this possible trend in sensitivity, a general trend in the sink strength may also affect the comparison between 2003 and 2018.

## 5. Conclusion

According to the presented estimates of net ecosystem exchange (NEE) in Europe based on atmospheric CO_2_ measurements, the heat and drought conditions in the summer of 2018 caused NEE anomalies in most parts of Europe, especially in Western, Central and parts of Northern Europe. Integrated spatially over Central and Western Europe, the 2018 summer NEE response was still only 42 to 75% of that during the heatwave of 2003. In Eastern Europe, NEE anomalies were detected in 2003 and 2018 as well, although they were smaller in absolute size than anomalies in 2010 and 2015.

Using NEE anomalies like that in 2018 as ‘natural experiments’, we estimated the sensitivities of NEE against variations in temperature and in water availability (here represented by the six-monthly accumulated Standardized Precipitation Evapotranspiration Index, SPEI06). This ‘NEE-T-W inversion’ confirms previous conclusions on the influence of temperature. To the extent that it meaningfully separates the effects of temperature and water availability, it additionally suggests that water availability affects NEE in the mid-latitudes directly through inhibition of photosynthesis during drought, and in high-latitude ecosystems through its covariation with incoming radiation.

Though these inverse estimates may be affected by considerable (but hard to quantify) errors particularly at the rather small sub-European scale considered here, the climate-based results of the NEE-T-W inversion and results based on explicit year-to-year degrees of freedom (standard inversion) tend to broadly agree on the domain and strength of many of the larger NEE anomalies. Despite the complex underlying mechanisms, this suggests that the momentary climate conditions play a substantial role in the interannual NEE variability.

The similarities of the standard and NEE-T-W inversion results at least in the largest anomalies further suggest that the relatively dense European atmospheric CO_2_ measurements indeed provide information on year-to-year NEE anomalies within European subregions, at least in summer.

## Data accessibility

The inversion results presented here are available for use in collaborative projects from the Jena CarboScope website http://www.BGC-Jena.mpg.de/CarboScope/ (to be identified by run ID, table 1).
